# The National Network of Nurses and Midwives

**Published:** 1921-07-16

**Authors:** 


### The National Network of Nurses and Midwives.

Considerable light is thrown upon the problems connected with rural nursing by the annual meetings which have recently taken place of County Nursing Associations. The Oxford Nursing Federation has been successful in attracting a good supply of candidates, and at the close of the year had no vacant posts save in one or two of the six newly formed centres. There are now fifty affiliated associations. The Shropshire Association now nearly covers the county, but still lacks sixteen associations to complete the midwifery service. The shortage of nurse-midwives is not quite so acute as it was, but many nurses are leaving. The Devonshire Nursing Association is also suffering much from nurses resigning—twenty in the past year, of whom nine are going to be married. Devon has 132 affiliated associations, covering 271 parishes. They have sent twenty-nine pupils for training—a large increase, counterbalanced, alas! by the resignations. The appointment of a second assistant superintendent has become necessary, as the present superintendent, Miss Bell, has long been doing the work of two people.

At Ottery St. Mary the County Nursing Superintendent recently told subscribers that when she came to Devonshire they had five nurses for eight parishes, whereas they now have 150 nurses. The Somerset County Nursing Association also reports an increase in the number of nurses employed from 118 to 165. When we are told of the terrible shortage of district nurses, should not this rapid increase be given its full value? Let us be reasonable and admit that the supply of candidates has largely increased, but not largely enough yet to meet all wants.

The Suffolk Nursing Association made a new departure last year by splitting into two divisions, for the east and west parts of the county respectively. East Suffolk employs forty-one nurses and is training eight, while six new associations are in process of formation. There are twenty-one districts affiliated in West Suffolk, in all of which health visiting is done by the nurse. Three nurses are in training, and several new centres have been formed.

There are still 364 parishes in Norfolk without any proper nurses at all, as against 322 in which nursing associations have been formed. Candidates are not forthcoming in sufficient numbers to be trained, and we regret to learn that the rule is for no candidates to be accepted from outside the county. At the annual meeting of the Norfolk Nursing Federation the Countess of Albemarle was in the chair, and the Countess of March spoke strongly on the need for placing nursing on a provident basis. She objected to the expression "nursing of the sick poor," and said these words ought to be scratched out of every circular dealing with the work. But, after all, when the upkeep of one nurse now costs a rural nursing association about £3 10s. a week, how is it possible among a small population on a contribution of one penny, or even twopence, a week to talk of a provident basis?

